# Pierre Saliou (1940-2024)

**DOI:** 10.48327/mtsi.v4i3.2024.566

**Published:** 2024-09-09

**Authors:** Yves BUISSON

**Affiliations:** Membre de l'Académie nationale de médecine; SFMTSI Société francophone de médecine tropicale et santé internationale (ancienne SPE), Hôpital Pitié-Salpêtrière, Pavillon Laveran, 47-83 Boulevard de l'Hôpital, 75651 Paris cedex 13, France

C'est avec une profonde tristesse que nous avons appris le décès de notre ami Pierre Saliou survenu le 3 février 2024 à l’âge de 84 ans.

**Figure 1:**
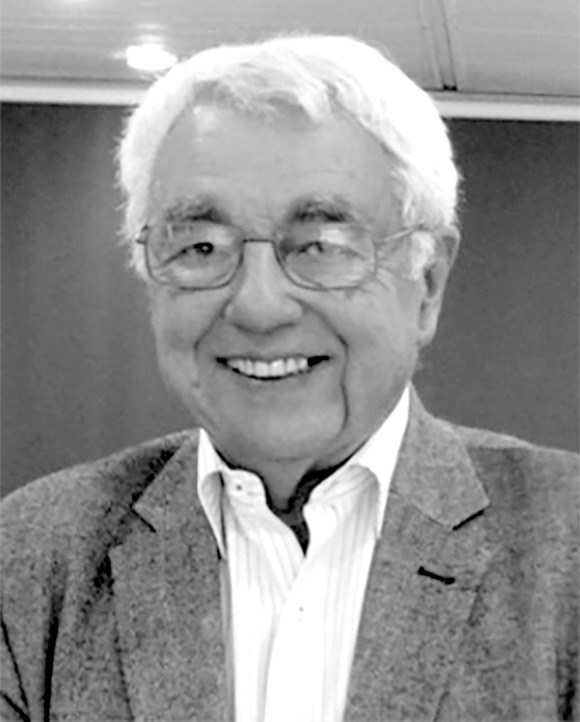
Pierre Saliou en 2016 aux XXII^e^ Actualités du Pharo (crédit photo : Patrice Milleliri)

**Figure 2:**
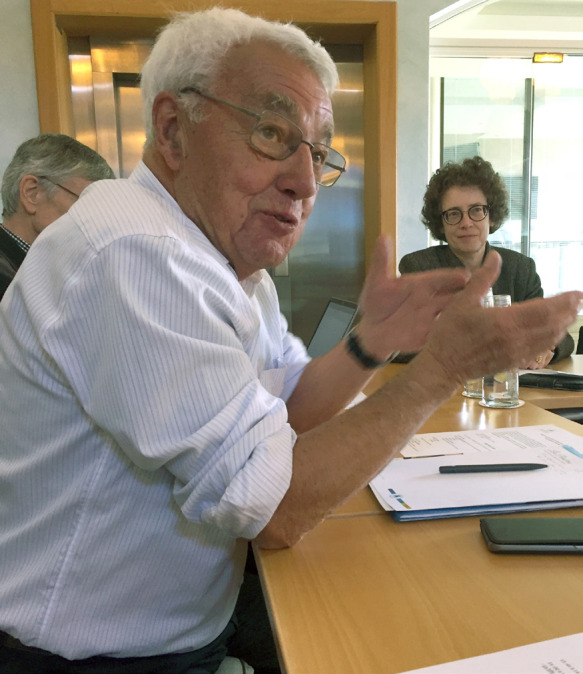
Pierre Saliou en 2019 lors du séminaire au Centre des Pensières, Fondation Mérieux, Veyrier-du-Lac (crédit photo : Alain Epelboin)

Pierre Saliou a été médecin militaire, biologiste, épidémiologiste, tropicaliste, coopérant, vaccinologue, enseignant, académicien… un parcours professionnel aussi riche que diversifié, qui devait le conduire sur les traces prestigieuses de Gaston Muraz, de Léon Lapeyssonnie et de Charles Mérieux. En ce jour du 10 février 2024, dans l’église du Val-de-Grâce, les nombreuses personnes qui s’étaient assemblées pour lui dire adieu portaient un témoignage individuel des multiples épisodes de sa carrière exceptionnelle.

Pierre est un breton. Il est fier de son patronyme finistérien, Saliou, dérivé du prénom biblique Salomon, qui suscitera souvent des confusions avec le prénom homophone sénégalais. Il est né et a grandi dans le Finistère, à l'ombre du viaduc de Morlaix. En 1958, il réussit le concours d'entrée à l’École du service de santé des armées de Lyon. Au cours de ses études, il est attiré par la parasitologie qu'il découvre au laboratoire de la faculté de médecine auprès des professeurs Jean Coudert et Jean-Paul Garin. Avec Pierre Ambroise-Thomas, récemment rapatrié d'Algérie, il étudie le diagnostic sérologique du paludisme humain par immunofluorescence. Ce sera le sujet de sa thèse qu'il soutient en 1964. Après le stage à l’École d'application du Val-de-Grâce à Paris, il reçoit sa première affectation comme médecin d'unité dans les Forces françaises en Allemagne, d'abord à Constance, puis à Baden-Baden. En 1969, il passe avec succès le concours de l'assistanat de biologie des hôpitaux des armées et complète sa formation à l'Institut Pasteur de Paris par le Grand cours 1969-1970 (microbiologie et immunologie) prolongé l'année suivante par le cours d’épidémiologie. En 1973, ayant réussi le concours de biologiste des hôpitaux des armées, il est détaché au ministère de la Coopération pour servir en République de Haute-Volta (futur Burkina Faso) à Bobo-Dioulasso, au Centre Muraz que dirige Jean-Henri Ricossé. Ce poste prestigieux est le siège de l'OCCGE (Organisation de coordination et de coopération pour la lutte contre les grandes endémies). Chef de la section de biologie, il découvre *in situ* la diversité de la pathologie tropicale ainsi que ses paroxysmes épidémiques lors des flambées de choléra et de méningite cérébrospinale. De retour en France en 1977, il est affecté au Laboratoire de biologie médicale de l'Hôpital d'instruction des armées (HIA) Bégin dirigé par André Thabaut et prépare le concours d'agrégation. En 1980, il est nommé professeur agrégé du Val-de-Grâce dans la chaire d’épidémiologie. L'année suivante, il rejoint le laboratoire de biologie médicale de l'HIA du Val-de-Grâce. Il y complète sa formation en anatomopathologie auprès du professeur Henri-Michel Antoine qu'il remplace en 1983 comme chef de service. L'année 1986 marque un grand virage dans sa carrière : il démissionne de l'armée avec le grade de Médecin-en-chef de 1^ère^ classe et prend les fonctions de directeur médical de Pasteur Mérieux sérums et vaccins. Ce groupe, créé l'année précédente par la fusion d'Institut Pasteur Production et de l'Institut Mérieux, changera plusieurs fois d'appellation (Pasteur-Mérieux Sérums & Vaccins, puis Pasteur Mérieux Connaught, puis Aventis Pasteur, puis Sanofi Pasteur). Pierre se lance alors avec passion dans la grande aventure des vaccins, en collaboration avec les meilleurs spécialistes de l’époque : Charles Mérieux, l'industriel qui voulait vacciner tous les enfants du monde, Philippe Stoeckel, le président fondateur de l'Agence de médecine préventive, et Stanley Plotkin, le père de la vaccinologie qui aimait à rappeler, s'agissant des spécialistes de la désinformation, qu'il n'existe pas de vaccin contre la stupidité.

**Figure 3:**
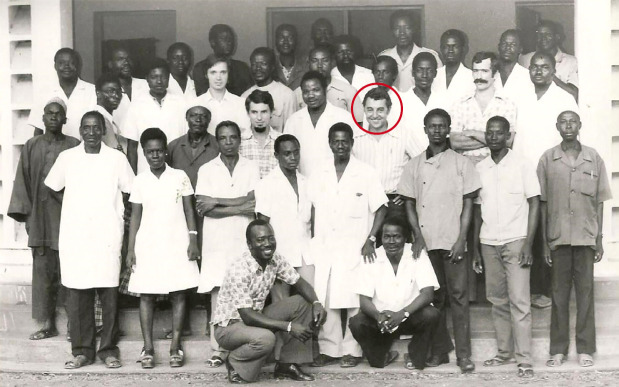
Laboratoire de biologie du Centre Muraz, 1976 (crédit photo: archive familiale)

Pierre s'implique dans de nombreuses initiatives mettant à profit sa triple expertise de biologiste, d’épidémiologiste et de vaccinologue : président (pendant plus de 10 ans) du Groupe d'expertise et d'information sur la grippe (GEIG), président (de 2004 à 2017) du Groupe d'intervention en santé publique et épidémiologie (GISPE) qui organise les Actualités du Pharo depuis la fermeture de l'Institut de médecine tropicale du service de santé des armées, et aussi membre du Groupe d’études en préventologie (GEP) fondé en 2009 par Jean-Louis Koeck pour gérer la base de données des recommandations vaccinales^1^1https://www.mesvaccins.net and https://www.medecinedesvoyages.net/.

L'agrégé du Val-de-Grâce gardera toute sa vie le goût d'enseigner. La vaccinologie est une science nouvelle dont il se fait l'apôtre, animé d'une infatigable ténacité. En 2006, il publie avec Jean-Jacques Bertrand et Bernard Seytre *Les sentinelles de la vie, le monde des vaccins,* un ouvrage dont le sous-titre exprime la question fondamentale : « Sommes-nous prêts à vacciner la planète ? ». En qualité de professeur visiteur de l'université de Bordeaux, il est coordonnateur pédagogique du Cours international francophone de vaccinologie (CIFV) créé en 2006 en partenariat avec le Service de santé des armées et avec le parrainage de la Société de pathologie exotique. Des sessions de ce cours sont délocalisées au Congo en 2007, au Niger en 2009, au Laos en 2012 et à l'Institut Pasteur d'Algérie en 2017. D'autres sessions sont organisées en 2006, 2008 et 2020 à l'Université Senghor d'Alexandrie dont il est professeur associé.

**Figure 4:**
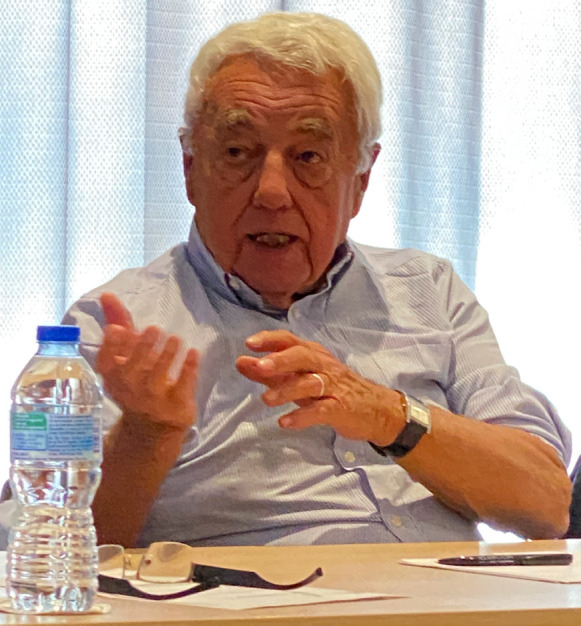
Pierre Saliou en 2021 lors du séminaire d'Aix en Provence, (crédit photo : Alain Epelboin)

**Figure 5:**
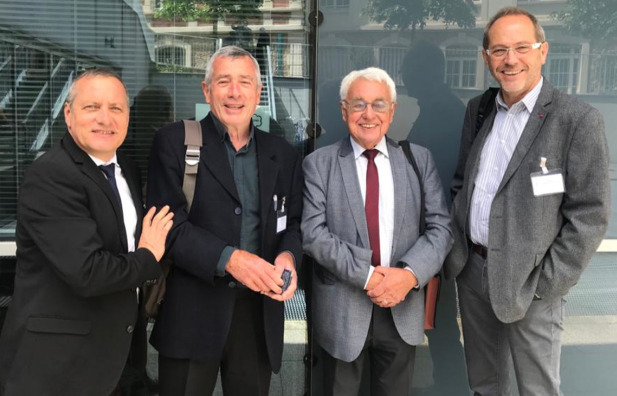
Bureau du GISPE à l'Institut Pasteur, 2020, de gauche à droite : Jean-Marie Milleliri, Jean-Loup Rey, Pierre Saliou, Jean-Paul Boutin (crédit photo : Patrice Milleliri)

De nombreuses associations accueillent Pierre en leur sein. Dès la fin du Grand Cours, il intègre l'Association des anciens élèves de l'Institut Pasteur (AAEIP) avant d'en devenir administrateur en 1998, puis président en 2015. Il rejoint aussi l'Association pour le développement de l’épidémiologie de terrain (EPITER) dont il devient membre d'honneur en 1991. Plus tard, en 2021, il est nommé membre d'honneur de l'Association des Anciens Combattants et Amis de la Légion étrangère (AACLE) avec Jean-Marie Milleliri à la suite d'un colloque international sur les vaccins contre la Covid-19. L'année suivante, il est reçu membre de l'académie de Vaucluse en Avignon. Pierre est aussi un membre actif dans plusieurs sociétés savantes : la Société française de médecine des armées (SFMA) pour laquelle il organise au Val-de-Grâce, de 1983 à 1996, des réunions communes avec le Club de bio-pathologie comparée, imaginé et créé par Charles Mérieux bien avant que la mode « *One health* » s'empare du concept. En 1995, il rejoint la *Société de médecine des voyages* (SMV) où il apporte son expertise de tropicaliste et de vaccinologue. Mais c'est la Société de pathologie exotique (SPE) qui, des années avant de devenir la SFMTSI, est son véritable port d'attache. Il est élu membre en 1977, dès son retour de Haute-Volta, la Société étant alors présidée par Lucien Brumpt. Il en sera élu administrateur, puis président de 2003 à 2006. À l'occasion du centenaire de l’École du Pharo en 2005, il organise avec Pierre Ambroise-Thomas le congrès international de la SPE à Marseille, en jumelage avec les congrès de la Fédération européenne des sociétés de médecine tropicale et santé internationale (ECTMIH) et de la Fédération internationale pour la médecine tropicale (ICTMM), réunissant au total plus de 1 000 participants. Outre les nombreuses journées thématiques conçues et préparées à son initiative, les 39 articles publiés de 1965 à 2023 dans le *Bulletin de la SPE* et listés ci-dessous témoignent d'une participation fidèle et assidue aux activités scientifiques de « la vieille dame centenaire », même après son rajeunissement en SFMTSI.

En 2004, Pierre est élu membre titulaire de l'Académie des sciences d'Outre-mer qu'il présidera en 2014. Très actif au sein de cette compagnie, il coordonne notamment la séance mémorable du 6 mars 2020 intitulée « Médecine et santé internationale, du passé à l'avenir » présidée par Marc Gentilini et dont les textes des interventions sont publiés dans *Mondes et cultures.*

De nombreuses distinctions jalonnent sa brillante carrière : Chevalier de la Légion d'honneur, Chevalier de l'Ordre national du Mérite, Chevalier des Palmes académiques et médaille d'argent pour travaux scientifiques et techniques du ministère de la Défense.

Qui se souvient de Pierre Saliou garde en sa mémoire un sourire inaltérable, expression d'un caractère avenant et d'une gentillesse innée. Ses collaborateurs et collaboratrices appréciaient particulièrement sa bonne humeur, son attention aux autres, sa bienveillance et ses qualités de médiateur. Par-delà le collègue chaleureux et sympathique, ses amis et ses proches avaient pu découvrir un être sensible et cultivé, amateur de théâtre, de musique et d'interminables discussions autour d'une bonne table. Quand le temps des malheurs est arrivé, nous avons tous été impressionnés par la force d’âme avec laquelle il a traversé les épreuves successives du décès de son épouse Nicole et de sa fille Isabelle. Malgré la peine qui le rongeait, il ne s'est jamais départi de son optimisme et de son désir d'aller de l'avant, affrontant avec un courage indéfectible la longue maladie qui devait l'emporter.

Pierre a maintenant trouvé une place légitime auprès de nos illustres aînés qui ont fondé et fait prospérer la SPE. Puisse son modèle inspirer et motiver nos jeunes successeurs pour assurer l'avenir de la SFMTSI.
