# Supplementary material for: Pierre Saliou (1940-2024)
Source: Med Trop Sante Int. 2024 Sep 9;4(3):mtsi.v4i3.2024.566. [Article in French] doi: 10.48327/mtsi.v4i3.2024.566 (PMC11809068; doi:10.48327/mtsi.v4i3.2024.566)
Supplement: Related texts

# Research article: Pierre Saliou (1940-2024)

We were deeply saddened to learn of the death of our friend Pierre Saliou on February 3, 2024, at the age of 84.

**Figure 1:** Pierre Saliou in 2016, at the 22nd edition of the Actualités du Pharo (photo credit: Patrice Milleliri)

**Figure 2:** Pierre Saliou in 2019 during the seminar at the Centre des Pensières, Fondation Mérieux, Veyrier-du-Lac (photo credit: Alain Epelboin)

Pierre Saliou was a military doctor, biologist, epidemiologist, tropicalist, aid worker, vaccinologist, teacher and academician… a career as rich as it was varied, which would lead him in the prestigious footsteps of Gaston Muraz, Léon Lapeyssonnie and Charles Mérieux. On this day, February 10, 2024, in the church of Val-de-Grâce, the many persons who had gathered to bid him farewell bore individual witness to the many episodes of his exceptional career.

**Figure 3:** Muraz Center Biology Laboratory, 1976 (photo credit: family archive)

Pierre was a Breton, proud of his Finistère surname, Saliou, derived from the biblical Salomon, often confused with the Senegalese homophone. He was born and raised in Finistère, in the shadow of the Morlaix viaduct. In 1958, he graduated from the École du Service de santé des armées in Lyon. During his studies, he was drawn to parasitology, which he discovered in the laboratory of the Faculty of Medicine under Professors Jean Coudert and Jean-Paul Garin. With Pierre Ambroise-Thomas, recently repatriated from Algeria, he studied the serological diagnosis of human malaria using immunofluorescence. This became the subject of his thesis, which he defended in 1964. After training at the École d'application du Val-de-Grâce in Paris, he received his first assignment as a unit doctor of the French Forces in Germany, first in Konstanz, then in Baden-Baden. In 1969, he passed the competitive examination for the position of assistant biologist at army hospitals, and completed his training at the Institut Pasteur in Paris with the Grand Cours 1969-1970 (microbiology and immunology), followed by the epidemiology course the following year. In 1973, having passed the competitive examination for army hospital biologist, he was seconded to the Ministry of Cooperation to work in the Republic of Upper Volta (later Burkina Faso) at Bobo-Dioulasso, at the Muraz Center headed by Jean-Henri Ricossé. This prestigious institute is the headquarters of the OCCGE (Organisation de coordination et de coopération pour la lutte contre les grandes endémies). As head of the biology section, he discovered *in situ* the diversity of tropical pathology and its epidemic paroxysms during outbreaks of cholera and cerebrospinal meningitis. Returning to France in 1977, he was assigned to the Medical Biology Laboratory of the Hôpital d'Instruction des Armées (HIA) Bégin, directed by André Thabaut, and prepared for the agrégation examination. In 1980, he was appointed professeur agrégé of epidemiology at Val-de-Grâce. The following year, he joined the Medical Biology Laboratory at HIA Val-de-Grâce. There, he completed his training in anatomopathology under Professor Henri-Michel Antoine, whom he succeeded as head of the department in 1983. 1986 marked a major turning point in his career: he retired from the army with the rank of 1^st^ class Chief Medical Officer and took up the post of Medical Director at Pasteur Mérieux Serums & Vaccines. This company, created the year before by the merger of Institut Pasteur Production and Institut Mérieux, changed its name several times (Pasteur-Mérieux Sérums & Vaccins, then Pasteur Mérieux Connaught, then Aventis Pasteur, today Sanofi Pasteur). Pierre plunged passionately into the great adventure of vaccines, working with the best specialists of the time: Charles Mérieux, the industrialist who wanted to vaccinate every child in the world; Philippe Stoeckel, the founding president of the Agence de médecine préventive; and Stanley Plotkin, the father of vaccinology, who liked to remind when talking about disinformation specialists, that there is no vaccine against stupidity.

Pierre was involved in numerous initiatives, drawing on his triple expertise as a biologist, epidemiologist and vaccinologist: President (for over 10 years) of the Groupe d'expertise et d'information sur la grippe (GEIG), President (from 2004 to 2017) of the Groupe d'intervention en santé publique et épidémiologie (GISPE), which has organized the Actualités du Pharo since the closure of the Institut de médecine tropicale du Service de santé des armées, and also a member of the Groupe d’études en préventologie (GEP) founded in 2009 by Jean-Louis Koeck to manage the database of vaccine recommendations^1^1https://www.mesvaccins.net and https://www.medecinedesvoyages.net/.

This agrégé of the Val-de-Grâce had a lifelong love of teaching. Vaccinology is a new science, and he was its tireless apostle. In 2006, together with Jean-Jacques Bertrand and Bernard Seytre, he published *Les sentinelles de la vie, le monde des vaccins,* a book whose subtitle expresses the fundamental question: “Are we ready to vaccinate the planet?” Visiting professor at the University of Bordeaux, he was the educational coordinator of the Cours International Francophone de Vaccinologie (CIFV), created in 2006 in partnership with the Service de santé des armées and with the sponsorship of the Société de Pathologie exotique. Sessions of this course were moved to Congo in 2007, Niger in 2009, Laos in 2012 and the Institut Pasteur d'Algérie in 2017. Other sessions were organized in 2006, 2008 and 2020 at Senghor University in Alexandria, where he was an associate professor.

**Figure 4:** Pierre Saliou in 2021 during the Aix en Provence seminar, (photo credit: Alain Epelboin)

**Figure 5:** GISPE Board at the Institut Pasteur, 2020, from left to right: Jean-Marie Milleliri, Jean-Loup Rey, Pierre Saliou, JeanPaul Boutin (photo credit: Patrice Milleliri)

Numerous associations welcomed Pierre into their ranks. As soon as the Grand Cours was over, he joined the Association des anciens élèves de l'Institut Pasteur (AAEIP), becoming a director in 1998, and then president in 2015. He also joined the Association pour le développement de l’épidémiologie de terrain (EPITER), becoming an honorary member in 1991. Later, in 2021, he was named honorary member of the Association des Anciens Combattants et Amis de la Légion étrangère (AACLE) with Jean-Marie Milleliri, following an international symposium on Covid-19 vaccines. The following year, he was elected to the Académie de Vaucluse in Avignon.

Pierre was also an active member of numerous learned societies, including the Société française de médecine des armées (SFMA), for which he organized joint meetings at Val-de-Grâce from 1983 to 1996 with the Club de biopathologie comparée, conceived and founded by Charles Mérieux long before the One Health paradigm. In 1995, he joined the Société de médecine des voyages (SMV), where he contributed his expertise as a tropicalist and vaccinologist. But the Société de pathologie exotique (SPE), years before it became the SFMTSI, was his real home. He was elected as member in 1977, on his return from Upper Volta, when Lucien Brumpt was President. He was elected Director and then President from 2003 to 2006. On the occasion of the centenary of the École du Pharo in 2005, he organized, together with Pierre Ambroise-Thomas, the international congress of the SPE in Marseille, in conjunction with the congresses of the European Federation of Tropical Medicine and International Health Societies (ECTMIH) and the International Federation for Tropical Medicine (ICTMM), which brough together more than 1,000 participants. In addition to the numerous theme days conceived and prepared on his initiative, the 39 articles published in the *Bulletin de la SPE* from 1965 to 2023 and listed below, bear witness to his faithful and assiduous participation in the scientific activities of “the centenarian old lady”, even after its rejuvenation as SFMTSI.

In 2004, Pierre was elected a full member of the Académie des sciences d'Outre-mer, of which he was President in 2014. A very active member of this association, he coordinated the memorable session of March 6, 2020 entitled “Médecine et santé internationale, du passé à l'avenir”, chaired by Marc Gentilini, whose papers were published in *Mondes et cultures.*

His brilliant career was marked by numerous honors: Chevalier de la Légion d'honneur, Chevalier de l'Ordre national du Mérite, Chevalier des Palmes académiques and a silver medal for scientific work from the French Ministry of Defense. Those who remember Pierre Saliou will treasure his unfailing smile, the expression of a pleasant character and his innate kindness. His colleagues especially appreciated his good humor, his attention to others, his kindness and his ability to mediate. In addition to a warm and friendly colleague, his friends and family had discovered a sensitive and cultured person, a lover of theater, music and endless discussions around a good table. When tragedy struck, we were all impressed by the strength with which he endured the successive hardships of life of the death of his wife Nicole and his daughter Isabelle. Despite the grief that consumed him, he never lost his optimism and desire to move forward, facing the long illness that was to take his life with unfailing courage. Pierre has now taken his rightful place among the illustrious elders who founded and built the SPE. May his example inspire and motivate our younger successors to ensure the future of SFMTSI.
